# METTL3-Regulated lncRNA SNHG7 Drives MNNG-Induced Epithelial–Mesenchymal Transition in Gastric Precancerous Lesions

**DOI:** 10.3390/toxics12080573

**Published:** 2024-08-06

**Authors:** Jiabei Jian, Yanlu Feng, Ruiying Wang, Chengyun Li, Lin Zhang, Ye Ruan, Bin Luo, Geyu Liang, Tong Liu

**Affiliations:** 1 Institute of Occupational Health and Environmental Health, School of Public Health, Lanzhou University, Lanzhou 730000, China; jianjb2023@lzu.edu.cn (J.J.); lin_zhang@lzu.edu.cn (L.Z.); ruany@lzu.edu.cn (Y.R.); luob@lzu.edu.cn (B.L.); 2 Qinghai Provincial Center for Disease Control and Prevention, Institute of Immunization Planning, Xining 810000, China; 220203810@aa.seu.edu.cn; 3 Gansu Provincial Center for Disease Prevention and Control, Lanzhou 730000, China; wry1185116185@163.com; 4 Department of Toxicology, School of Public Health, Lanzhou University, Lanzhou 730000, China; lichengyun@lzu.edu.cn; 5 Key Laboratory of Environmental Medicine Engineering, Ministry of Education, School of Public Health, Southeast University, Nanjing 210096, China; lianggeyu@163.com

**Keywords:** METTL3, MNNG, SNHG7, gastric cancer, m6A RNA methylation

## Abstract

As a representative item of chemical carcinogen, MNNG is closely associated with the onset of gastric cancer (GC), where N6-methyladonosine (m6A) RNA methylation is recognized as a critical epigenetic event. In our previous study, we found that the m6A modification by methyltransferase METTL3 was up-regulated in MNNG-exposed malignant GES-1 cells (MC cells) compared to control cells in vitro, and long non-coding RNA SNHG7 as a downstream target of the METTL3. However, the functional role of METTL3 in mediating the SNHG7 axis in MNNG-induced GC remains unclear. In the present study, we continuously investigate the functional role of METTL3 in mediating the SNHG7 axis in MNNG-induced GC. RIP-PCR and m6A-IP-qPCR were used to examine the molecular mechanism underlying the METTL3/m6A/SNHG7 axis in MNNG-induced GC. A METTL3 knockout mice model was constructed and exposed by MNNG. Western blot analysis, IHC analysis, and RT-qPCR were used to measure the expression of METTL3, SNHG7, and EMT markers. In this study, we demonstrated that in MNNG-induced GC tumorigenesis, the m6A modification regulator METTL3 facilitates cellular EMT and biological functions through the m6A/SNHG7 axis using in vitro and in vivo models. In conclusion, our study provides novel insights into critical epigenetic molecular events vital to MNNG-induced gastric carcinogenesis. These findings suggest the potential therapeutic targets of METTL3 for GC treatment.

## 1. Introduction

Gastric cancer (GC), a type of malignant tumor found in the gastrointestinal tract, is a serious health threat with high rates of morbidity and mortality [1]. Recent data from the International Agency for Research on Cancer (IARC) indicates that approximately 990,000 individuals worldwide received a diagnosis of GC in 2022, resulting in roughly 740,000 fatalities [2]. Despite advances in diagnosis and treatment, GC patients still have a low 5-year survival rate, resulting in a poor prognosis for the majority [3,4,5]. Indeed, searching for biomarkers with high sensitivity and specificity has been a longstanding challenge in the early diagnosis of GC. The methods currently used for diagnosing GC, endoscopy, and biopsy are invasive and unsuitable for widespread screening [6]. This highlights the need for more accessible and less intrusive diagnostic options. 

According to pathological studies, gastric mucosal cell carcinogenesis is a slow and intricate process that progresses through multiple stages, including precancerous lesions of Gastric Cancer (PLGC), intramucosal carcinoma, and invasive carcinoma, with PLGC being a crucial pathological stage, significantly amplifying the possibility of GC. The occurrence of GC involves a range of genetic, epigenetic, epi-transcriptomic, and environmental changes. The pathogenesis of GC was linked to significant environmental chemical carcinogens. N-methyl-N-nitro-N-nitroguanidine (MNNG), a synthetic nitroso compound (NOC), is known to cause severe damage to cellular DNA, leading to various lesions that can result in gastric cancer [7,8]. MNNG is often used in scientific research to replace NOCs in nature and investigate its role in GC formation. In our previous studies, we focused on the potential role of epigenetic variation in MNNG-induced malignancy in gastric mucosal epithelial cells, revealing the paramount role of m6A RNA methylation in this process [9,10,11,12,13]. However, the critical molecular timing and the underlying mechanism of action of MNNG in gastric carcinogenesis remain unresolved. 

To investigate the regulatory effect of m6A modification in MNNG-induced GC, we previously identified the long non-coding RNA SNHG7 as a downstream target of the m6A modification methyltransferase METTL3 by performing methylated RNA immunoprecipitation sequencing (MeRIP-seq) [12]. LncRNAs play a crucial role in regulating GC occurrence, development, and metastasis. For instance, the lncRNA HNF1A-AS1 contributes to GC cell proliferation by promoting the degradation of P21 and bolstering the expression of cell cycle regulators [14]. In addition, the expression of the lncRNA FEZF1-AS1 was up-regulated in GCs, promoting the tumorigenicity of tumor stem cells through the FEZF1-AS1/miR-363-3p/HMGA2 axis [15]. Similarly, studies also revealed that lncRNA FERO and ROR could promote GC progression [16,17]. While our previous findings indicated that METTL3 influences the m6A methylation level of the lncRNA SNHG7, suggesting that SNHG7 could be a significant prognostic marker in GC progression, the underlying mechanisms and biological roles of SNHG7 in GC development due to MNNG exposure require further investigation. 

In the present study, based on previous findings, we established in vitro and in vivo models to continuously investigate the functional role of METTL3 in mediating the SNHG7 axis in MNNG-induced GC. These findings demonstrate that SNHG7 has a tumor-promoting role in MNNG-induced GC. SNHG7 is a target molecule of METTL3, which influences cellular epithelial–mesenchymal transitions (EMTs). This discovery provides a deeper understanding of the molecular mechanisms involved in GC progression and suggests potential targets for therapeutic intervention.

## 2. Materials and Methods

### 2.1. Cell Lines and Cell Culture

The MMNG-induced malignantly transformed cells derived from GES-1 cells (MC cells) were provided by the Key Laboratory of Environmental Medicine Engineering, Ministry of Education, School of Public Health, Southeast University (Nanjing, China). Our previous study describes the specific procedures for MMNG-induced malignant transformation cells [12,13]. The GES-1 cell was briefly treated with long-term exposure to MNNG (Sigma-Aldrich, St. Louis, MO, USA). The MNNG was dissolved in DMSO to create a stock solution with a 1.0 mmol/L concentration. Cells were exposed to MNNG at a concentration of 5 × 10^−5^ mol/L for 24 h in the dark for each passage. Following exposure to MNNG, the cells were grown in a regular RPMI-1640 medium, and the medium was replaced every 48 h. The exposure process lasted for a duration of 20 weeks, during which the cells underwent 40 passages (MC-40), ultimately leading to the development of a malignant phenotype.

The GC cell line (HGC-27, AGS) with STR certification was purchased from Guangzhou Cellcook Biotech Co., Ltd (Guangzhou Cellcook Biotech Co., Ltd, Guangzhou, China). The cells were cultured at 37 °C in a 5% CO_2_ humidified incubator using RPM-1640 media (Thermo Fisher Scientific, Waltham, MA, USA) supplemented with 10% fetal bovine serum, 100 μg/mL Penicillin, and 100 μg/mL Streptomycin.

### 2.2. Lentiviral Packaging and Cell Transfection

Hanbio Biotechnology Co., Ltd. (Wuhan, China) designed and commercially synthesized METTL3 knockdown lentiviruses and SNHG7 overexpression lentiviruses (Supplementary Figure S1). Confirmed METTL3 shRNA targeting sequences and the reference gene are listed in Supplementary Table S1. Plasmids were transfected into MC-40 and AGS cells using the Lipofectamine 2000 kit (Invitrogen, Carlsbad, CA, USA), and cells transfected with NC vector were used as controls. Puromycin was used to select stable clones. The green fluorescent protein (GFP) production area was assessed using ImageJ software (1.54 h 15 December 2023), and transfection efficiency was estimated by the percentage of GFP green fluorescence (ZsGreen) cells. To calculate the percentage of GFP-positive cells, the authors should count all cells and GFP-positive cells in one image, then calculate the percentage of GFP-positive cells using this formula: GFP-positive cells/all cells*100%. The RT-qPCR and Western blot analysis detected the expression level of METTL3 of stable transfection cell lines, which confirmed the gene silencing.

### 2.3. RNA Total m6A Quantification

The EpiQuik™ m6A RNA Methylation Quantification Kit (Colorimetric) was obtained from the Epigentek Group Inc. (Farmingdale, NY, USA), which measured the total RNA m6A methylation quantification level. Briefly, 200 ng RNA was injected into the assay wells. Then, the m6A levels of each well were quantified colorimetrically by reading the absorbance at a wavelength of 450 nm. 

### 2.4. Construction of METTL3 Knockout Mice Model and Exposed by MNNG

Wild-type (WT) and Mettl3 knockout (KO) transgenic mice on C57BL/6JGpt were purchased from Gem-Pharmatech Co., Ltd. (Nanjing, China). All mice were genotyped by agarose gel electrophoresis and included in the appropriate subgroups. KO mice were those with successful knockout of METTL3 (+/−), and WT mice were the normal wild-type mice. Due to the homozygous offspring of METTL3 knockout mice being embryonic lethal (NCBI: http://www.informatics.jax.org/marker/MGI:1927165, accessed on 13 January 2023), the METTL3 knockout mice (KO mice, +/−) used in the present study were heterozygous mice.

For MNNG exposure, 6–8-week-old male KO and WT mice were exposed to MNNG for 6~18 weeks. Specific induction methods and procedures are described in our previous study [13]. Briefly, 42 KO and WT mice were randomly allocated to four groups (6–8/each group) as follows: (1) mice fed with drinking water; (2) mice fed with drinking water dissolved with MNNG (150 µg/mL) and 1‰ DMSO; (3) METTL3-KO mice fed with drinking water; and (4) METTL3-KO mice fed with drinking water dissolved with MNNG (150 µg/mL) and 1‰ DMSO for 18 weeks. All the mice were housed in a pathogen-free, temperature-controlled environment under a 12 h light/dark cycle. Immunohistochemistry (IHC) analysis was conducted to measure METTL3 and EMT marker protein expression. 

### 2.5. Immunohistochemistry (IHC) Analysis 

The IHC protocol was conducted by Service Bio-tech Co., Ltd. (Wuhan, China) [18]. Paraffin embedding was performed on the collected samples, which were then sectioned into 4 mm slices. The slices were subjected to antibody probing overnight at 4 °C using antibodies against METTL3 (Abcam195352) and epithelial–mesenchymal transition (EMT) marker proteins (Abcam, Cambridge, UK). Then, the sections were washed thrice with PBS and incubated with a biotin-labeled secondary antibody (Abcam) for 10 min. 

Subsequently, the sections were washed with streptavidin-peroxidase and incubated for 10 min. The sections were treated with the DAB Substrate Kit (MXB, Fuzhou, China, DAB-0031) to visualize the immunohistochemical staining. Scan images of the immunohistochemical sections were acquired using a tissue section digital scanner (Eclipse Ci-L, Nikon, Japan) or imaging system (CaseViewer2.4, 3DHISTECH, Budapest, Hungary), and tissue measurements were automatically obtained using the Sevier Image Analysis System.

### 2.6. Cell Proliferation Assays

Briefly, the cells were seeded in a 96-well plate at a density of 3 × 10^3^ cells per well and incubated in a CO_2_ incubator at 37 °C for 0 h, 24 h, 48 h, and 72 h. Subsequently, 10 μL of the Cell Counting Kit-8 mixture (Beyotime Institute of Biotechnology, Shanghai, China) was added to each well, and the plate was further incubated for 2 h. The absorbance of the samples was then measured at a wavelength of 450 nm using a spectrophotometer.

### 2.7. Migration and Invasion Assays

After 24 h of incubation, cells at a density of 1 × 10^5^ cells per well were seeded in the upper chambers of the Transwell plates in a serum-free medium. The upper chambers were coated with Matrigel (BD Biosciences, Bergen County, NJ, USA) for the invasion assay. Following an additional 24 or 48 h of incubation, the cells that had migrated or invaded the bottom surface of the membranes were fixed and stained using a 1 mg/mL crystal violet solution. The migrated or invaded cells were then counted using the FSX100 microscope (Olympus, Tokyo, Japan).

### 2.8. RNA Isolation and Quantitative Real-Time PCR (RT-qPCR)

Briefly, RNA was extracted and purified using the TRIzol reagent (Invitrogen, Carlsbad, CA, USA), and the concentration was determined using the NanoDrop 2000 spectrometer (Thermo Fisher Scientific, Waltham, MA, USA). The two-step reverse transcription process was performed to convert RNA into cDNA, followed by real-time PCR using the StepOneplus real-time PCR system (Applied Biosystems, Carlsbad, CA, USA) to detect the expression of the target gene. All mRNA and lncRNA primers were purchased from General Biotech Co., Ltd. (Shanghai, China), and reverse transcription of mRNA was conducted with the A214 Reverse Transcription System Kit (GenStar, Beijing, China) using random primers. The primer sequences for the target gene and housekeeping genes are listed in Table 1. The comparative Ct method was used for the fold-change, and the data were analyzed using the relative 2^−ΔΔCt^ method. Used primers are presented in Table 1.

### 2.9. Western Blot Analysis

Briefly, proteins were extracted from extracts from cells and tissue samples, and the concentration was determined using a BCA kit and then mixed with ultrapure water and 5× SDS buffer. Protein extracts were disintegrated using 10% SDS-PAGE and then transferred to the nitrocellulose membrane (Millipore, Billerica, MA, USA). After blocking with 5% milk in TBST for 2 h, the membranes were incubated with primary antibodies (METTL3 ab195352, 1:1000; GAPDH ab181602) overnight at 4 °C. Then, the membranes were incubated with a secondary antibody for another 1.5 h. Each band was tested using the ECL chromogenic kit (Thermo Fisher, Scotts Valley, CA, USA) and visualized with the Tanon-5200 chemiluminescence imaging system (Tanon Science & Technology, Shanghai, China). The intensities of the target protein were normalized to the intensity of the control protein GAPDH when processing the results. 

### 2.10. m6A-Modified RNA Immunoprecipitation qPCR (m6A IP-qPCR)

m6A-IP-qPCR was conducted following the instructions provided with the m6A-IP Kit from Cloud-Seq Biotech (Shanghai, China). For m6A-qPCR, m6A-enriched RNA was reverse-transcribed using the cDNA Reverse Transcription Kit (Thermo Fisher Scientific), and 15% of the total RNA served as the input sample. The RNA was subjected to immunoprecipitation with an anti-m6A antibody (Abcam) in the immunoprecipitation buffer, followed by incubation with protein A/G magnetic beads overnight at 4 °C. Finally, RT-qPCR analysis was performed to measure the proportion of input RNA and immunoprecipitated m6A RNA.

### 2.11. RNA-Binding Protein Immunoprecipitation (RIP) Assay

According to the manufacturer’s protocol, RIP assay was performed using the Magna RIPRNA-Binding Protein Immunoprecipitation Kit (Millipore, Billerica, MA, USA). Briefly, after formaldehyde-crosslinking (0.3% for 10 min), 2 × 10^7^ cells were lysed in RIP-lysis buffer. Magnetic Bead Protein A/G was incubated with an anti-METTL3 rabbit antibody (ab195352, Abcam) or normal rabbit IgG for 1 h at room temperature. Then, the coated beads were incubated with prepared cell lysates overnight at 4 °C. Then, the RNA was extracted and resuspended in 10 mL of RNase-free water. The RNAs were analyzed by RT-qPCR.

### 2.12. Statistical Analyses

All biological experiments were performed three times independently. Continuous variables were presented as mean values with their corresponding standard deviations. An analysis of variance (ANOVA), a Chi-square test, or non-parametric tests were employed as appropriate for data comparisons. A data analysis was performed using IBM SPSS Statistics 22.0 (SPSS Inc., Chicago, IL, USA) and GraphPad Prism 8.0 (La Jolla, CA, USA). Statistical significance was defined as *p* < 0.05.

## 3. Results

### 3.1. Subsection

#### 3.1.1. Knockdown of METTL3 Expression in MC-40 and GC Cells

In our previous finding, we revealed that METTL3 was indeed elevated in MNNG-exposed malignant GES-1 cells compared to control cells in vitro [13] and precisely targeted SNHG7, a critical lncRNA regulated by METTL3 via m6A modification in MNNG-induced GC [12]. To further explore the potential implication and functional role of METTL3 on SNHG7 in MNNG-induced GC, herein, we first knocked down the METTL3 expression in MNNG-induced 40-generation malignantly transformed Ges-1 cells (MC-40) and AGS GC cells by shRNA. 

**Figure 1:**
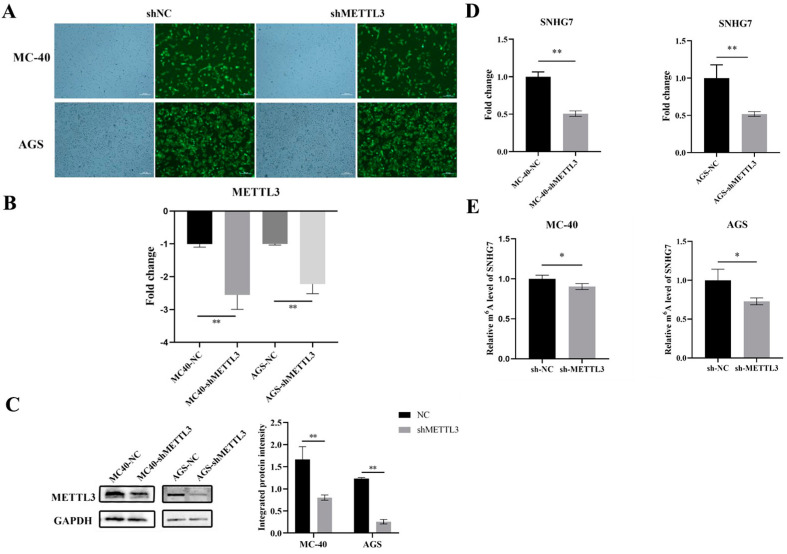
METTL3 increases m6A modification and expression of SNHG7 lncRNA. (**A**) Efficiency of lentiviral transfection in MC-40 and AGS cells. (**B**) The shRNA-mediated METTL3 repression was confirmed by qRT-PCR after lentivirus infection in MC-40 and AGS cells. (**C**) Western blot analysis of METTL3 expression in MC40-shMETTL3 and AGS-shMETTL3. (**D**) The lncRNA expression level of SNHG7 in MC-40 and AGS cells was confirmed by qRT-PCR after lentivirus infection. (**E**) The SNHG7 m6A modification levels in METTL3 knockdown GC cells were determined by m6A-IP-qPCR. * *p* < 0.05, ** *p* < 0.01.

By constructing a lentiviral vector, a stable knockdown of METTL3 was achieved. After the puromycin screening, the GFP area of stable transfection cell lines reached 85% for MC-40 cell and 95% for AGS cell (Figure 1A). The infection efficiency was confirmed via RT-qPCR and Western blot analysis. Compared with the negative control group (MC-40-shNC, AGS-shNC), the mean METTL3 mRNA expression level of MC-40-shMETTL3 and AGS-shMETTL3 were down-regulated 2.56-fold and 2.25-fold, respectively (*p* < 0.01) (Figure 1B). In addition, the results of the Western blot analysis also showed that the METTL3 knockdown cell groups (MC-40-shMETTL3 and AGS-shMETTL3) had significantly lower METTL3 protein expression levels (Figure 1C).

#### 3.1.2. Down-Regulation of METTL3 Inhibited lncRNA SNHG7 Expression 

Next, we investigated the regulatory mechanism of SNHG7 expression by METTL3. Consistently with our hypothesis, both MC-40 and AGS with METTL3 knockdown displayed an approximately 50% lower expression of SNHG7 (Figure 1D). For further validation, m6A-IP-qPCR was performed in MC-40 and AGS cells with METTL3 knockdown. The results demonstrated that the down-regulation of METTL3 decreased the immunoprecipitation of SNHG7 with anti-m6A antibodies in both cell lines (Figure 1E). 

#### 3.1.3. Overexpression of SNHG7 Reversed the Effect of Down-Regulation of METTL3 on the Proliferation, Migration, Invasion, and EMT of MC-40 and AGS Cells In Vitro

**Figure 2:**
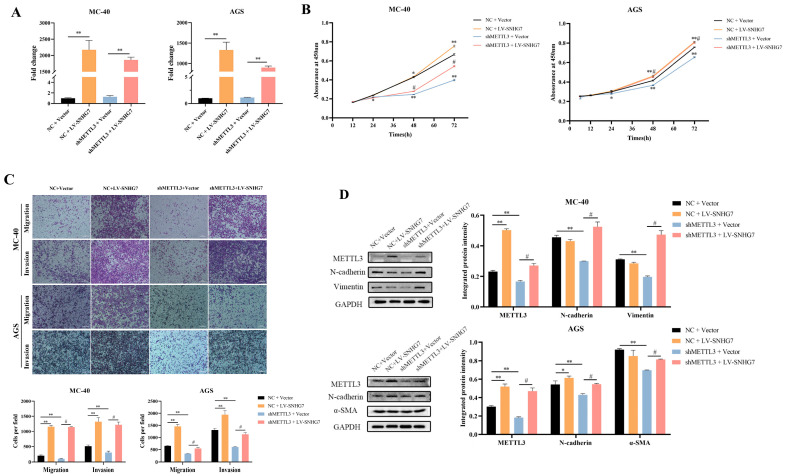
Overexpression of SNHG7 promoted tumorigenesis in vitro. (**A**) Overexpression of SNHG7 was confirmed by RT-qPCR in MC-40-shMETTL3 and AGS-shMETTL3 cells. (**B**) Overexpression of SNHG7 promoted the proliferation of MC-40 and AGS cells by CCK-8 assays. (**C**) METTL3 knockdown inhibited the migration and invasion ability of MC-40 and AGS cells, and overexpression of SNHG7 recovered the ability. (**D**) Western blot analysis of METTL3 and EMT markers in different cell lines. Compared with NC+Vector group, * *p* < 0.05, ** *p* < 0.01; compared with shMETTL3+Vector group, # *p* < 0.05.

Next, we overexpressed SNHG7 in two groups (MC-40 and AGS) of down-regulated METTL3 and paired control cells and confirmed the efficiency of overexpression by RT-qPCR (Figure 2A). The cck-8 assay showed that the down-regulation of METTL3 significantly inhibited the proliferation of MC-40 and AGS GC cells, whereas the overexpression of SNHG7 partially alleviated the decreased proliferative capacity caused by METTL3 knockdown (Figure 2B). Additionally, suppressing METTL3 markedly reduced the ability of MC-40 and GC AGS cells to migrate and invade. However, increasing the levels of SNHG7 restored their migratory and invasive capacities following METTL3 suppression (Figure 2C).

In addition, SNHG7 overexpression significantly enhanced the proliferation, migration, and invasion ability of MC-40 and AGS cells (Figure 2A–C).

After initial research suggested that cellular EMT plays a significant role in tumorigenesis caused by MNNG, it was necessary to investigate further the potential impact of METTL3 on MNNG-induced GC and its regulatory effects on the SNHG7 axis, specifically regarding EMT [13]. Impressively, the protein expression levels of EMT marker N-cadherin, Vimentin, and α-SMA were dramatically reduced after METTL3 depletion of MC-40 and AGS cells (Figure 2D). Reciprocally, the overexpression of SNHG7 partly augmented the EMT marker protein above (Figure 2D). These findings revealed that the overexpression of SNHG7 partly reversed the effect of the down-regulation of METTL3 on the proliferation, migration, invasion, and EMT of MC-40 and AGS cells in vitro.

#### 3.1.4. MNNG Exposure Exhibited a Significant Reduction in Gastric Weight, Body Weight, and Gastric Histopathological Changes in Mice

**Figure 3:**
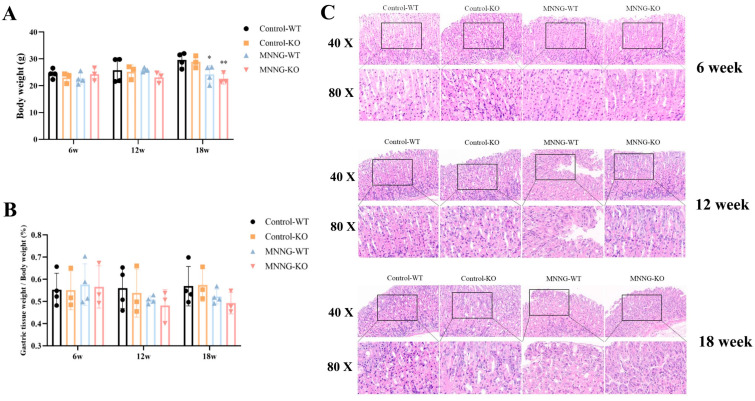
MNNG induces reduction in gastric weight, body weight, and gastric histopathological changes. (**A**) The body weight variations in MNNG-exposed rats; (**B**) the percent gastric tissue weight was measured in METTL3-KO mice and WT mice; (**C**) HE staining of gastric tissues from mice. * *p* < 0.05, ** *p* < 0.01. Control-WT vs. MNNG-WT; Control-KO vs. MNNG-KO.

To better understand the epi-transcriptome role of METTL3 in MNNG-induced EMT, we generated KO mice with systematic METTL3 deletion. Mouse genotypes (WT, KO type) were identified by agarose gel electrophoresis [13]. Mice exposed to MNNG demonstrated a reduced body weight more than controls after 18 weeks, and this weight loss was significantly enhanced in the METTL3-KO mice group (Figure 3A). Consistently with the trend of body weight loss, the percentage weight of gastric tissues of WT mice exposed to MNNG also decreased significantly during weeks 12–18 (Figure 3B). In contrast with controls, the gastric mucosa of mice exposed to high doses of MNNG (150 μg/mL, 18 weeks) displayed signs of inflammation and vascular bruising in the lamina propria and prominent granulocyte infiltration, and these changes were more pronounced in the MNNG-WT group (Figure 3C).

#### 3.1.5. Chronic Exposure to MNNG Leads to METTL3 Up-Regulation and Increases m6A Levels in the Peripheral Plasma of Mice

**Figure 4:**
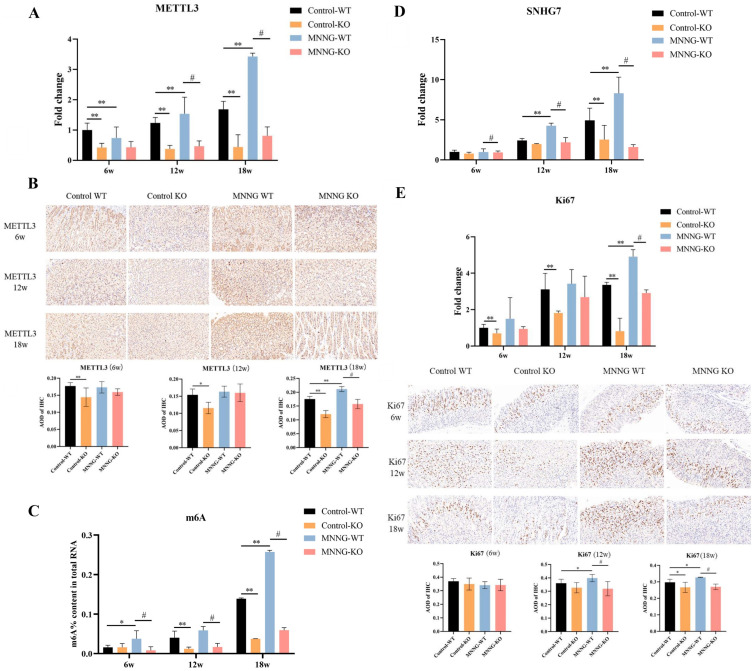
MMNG exposure elevates the levels of METTL3, SNHG7, m6A, and Ki67 in transgenic mice. (**A**) The mRNA levels of METTL3 in gastric tissues were determined by RT-qPCR after MNNG exposure. (**B**) Representative images of IHC staining for METTL3 protein of gastric tissue from transgenic mice. (**C**) The global content of m6A was examined by RNA methylation quantification assay. (**D**) The lncRNA expression levels of SNHG7 of gastric tissues with MNNG exposure. (**E**) The mRNA levels of Ki67 and representative IHC staining of Ki67 of gastric tissues with MNNG exposure. Compared with Control-WT group, * *p* < 0.05, ** *p* < 0.01; compared with MNNG-WT group, # *p* < 0.05.

Given the observed increase in METTL3 levels in malignant GES-1 cells exposed to MNNG [13], we validated whether exposure to MNNG induces the overexpression of METTL3 and alters relative m6A levels in plasma through in vivo experiments. RT-qPCR and HIC analysis firstly verified that METTL3 mRNA and protein expression was decreased in the Control-KO group of mice compared to the Control-WT group (Figure 4A,B, *p* < 0.01). In addition, consistently with our hypothesis, 18 weeks of MNNG exposure led to dramatically increased levels of METTL3 mRNA expression level in gastric tissues of WT mice (Figure 4A). The IHC results also demonstrated that METTL3 protein expression significantly increased in the MNNG-WT mice group compared to the Control-WT group (Figure 4B, *p* < 0.01). 

Subsequently, we investigated whether MNNG exposure could affect the m6A modification level by using the m6A RNA methylation quantification kit. After MNNG exposure, the MNNG-WT mice group also observed an increased total m6A level of total RNA in plasma (Figure 4C). Impressively, we also noticed that the knockout of METTL3 inhibited the increase in total RNA m6A levels caused by MNNG exposure, suggesting that METTL3 is responsible for total RNA m6A levels during MNNG exposure. 

#### 3.1.6. Time-Dependent Increase in the Expression of lncRNA SNHG7 and Cell Proliferation Indicator Ki67 in METTL3-KO/WT Mice Exposed to MNNG

The above in vitro results showed that SNHG7 is a downstream target of METTL3 regulation via m6A during the malignant transformation of MNNG-induced cells. The expression level of SNHG7 in METTL3 knockout mice after prolonged MNNG exposure was further measured to address this regulatory axis. The depletion of METTL3 significantly decreased the expression level of SNHG7 in METTL3-KO mice (Figure 4D). Furthermore, METTL3 deletion suppresses the stimulatory effects of MNNG on SNHG7 expression (Figure 4D). The qPCR analysis further confirmed that MNNG exposure induced the up-regulation of SNHG7 (Figure 4D). We further assessed Ki67 protein expression by IHC, a nuclear antigen in proliferating cells [19]. The results indicated that the cell proliferation indicator Ki67 increased in the Mettl3-KO and WT mice exposed to MNNG (Figure 4E). In addition, the depletion of METTL3 significantly inhibited the effects of MNNG on Ki67 expression (Figure 4E).

#### 3.1.7. Knockout of METTL3 Suppressed EMT Induced by MNNG Exposure in the Mettl3-KO/WT Mice

**Figure 5:**
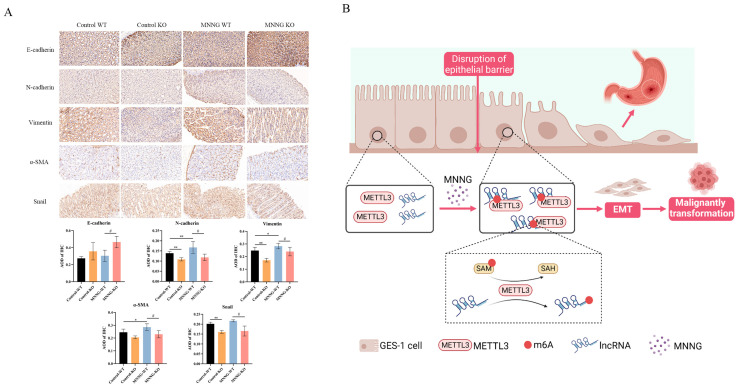
Representative images of IHC staining for EMT markers protein in gastric tissues of transgenic mice with MNNG exposure and research mechanism hypothesis. (**A**) IHC staining for EMT marker protein after 18 weeks of MNNG exposure (40×). Compared with Control-WT, * *p* < 0.05, ** *p* < 0.01; compared with MNNG-WT, # *p* < 0.05. (**B**) Research mechanism hypothesis.

Our previous studies revealed that the EMT process is vital in MNNG-induced malignancy [13]. Therefore, we next evaluate whether the knockout of METTL3 suppresses EMT induced by MNNG exposure (Supplementary Figure S2, Figure 5). Consistently with our previous hypothesis, 18 weeks of MNNG exposure led to increased levels of EMT markers, including N-cadherin, Vimentin, α-SMA, and Snail protein, in WT mice (Figure 5A). On the other hand, the depletion of METTL3 in METTL3-KO mice resulted in reduced levels of N-cadherin, Vimentin, α-SMA, and Snail protein while promoting the increased expression of the E-cadherin protein (Figure 5A).

## 4. Discussion

Previous studies have shown that m6A modification affected the processing, splicing, translation, stability, export, and polyadenylation of transcripts to regulate gene expression [20], which is associated with the progression of various diseases, especially tumors [21,22,23]. In the present study, we demonstrated that in MNNG-induced GC tumorigenesis, the m6A modification regulator METTL3 facilitates cellular EMT and biological functions through the m6A/SNHG7 axis using in vitro and in vivo models (Figure 5B).

NOCs were recognized as potential carcinogens influencing human health through various channels, including dietary, industrial, and agricultural consumer products. MNNG was particularly noteworthy due to its association with gastric pre-cancer and GC following chronic exposures [24,25,26,27]. As one of the most prevalent and studied post-transcriptional modifications found in mRNAs [28] and lncRNAs [29], m6A modification plays a role in a variety of cancers [30]. The m6A modification undergoes dynamic and reversible regulation by methyltransferases (writers), demethylases (erasers), and m6A-binding proteins (readers) [31,32]. 

Our previous studies found that METTL3, a major methyltransferase of RNA m6A modification, promotes GC progression through the METTL3/m6A/miR-1184/TRPM2 axis in MNNG-induced GC cells in GC [13]. METTL3 is involved in biological processes such as cell differentiation, proliferation, and migration, and it plays a role in many tumor types by mediating RNA m6A modification [32,33]. In addition, METTL3 can act on its downstream target YAP1 (a significant effector of the Hippo pathway), thereby altering the YAP1 pathway and promoting GC progression [34]. Accumulating evidence revealed that METTL3 regulates lncRNAs through m6A modification in various tumors [35,36,37]. It was reported that METTL3 regulates HDGF in an m6A-mediated manner to promote tumor angiogenesis and glycolysis and also regulates ZMYM1, SPHK2, and MYC to promote GC tumor metastasis [38,39,40]. In addition, Jin et al. found that METTL3 enhanced the stability of lncRNA MALAT1 in lung cancer, and MALAT1 sponged miR-1914-3p via YAP to promote invasion and metastasis of NSCLC cells [41]. Similarly, by me-RIP sequencing analysis, we previously identified the lncRNA SNHG7 as a potential downstream target of METTL3 in an m6A-dependent manner [12]. Therefore, in the present study, we further explored the biological functions and potential mechanisms of the METTL3-lncRNA SMHG7 axis in MNNG-exposed malignant gastric mucosal epithelial cells and the METTL3 knockout transgenic GC mouse model. 

A previous study revealed that m6A transferase METTL3 installed the m6A modification and enhanced the stability of SNHG7 in diabetic retinopathy [42]. Liu et al. also found that METTL3-mediated m6A modification of SNHG7 and enhanced its stability accelerates glycolysis in prostate cancer [43]. The above findings imply that SNHG7 may be a potential downstream regulatory molecule for METTL3. In this study, we observed that METTL3 knockdown inhibited SNHG7 expression and decreased the immunoprecipitation binding of SNHG7 to m6A antibodies in MC-40 and AGS cells. 

LncRNA SNHG7 is a novel vital oncogenic lncRNA widely reported in various cancers, and it is involved in several types of cancers, such as lung adenocarcinomas, pancreatic cancers, and bladder cancers [44,45,46]. SNHG7 acts as a sponge for miR-2682-5p to enhance the expression of ELK1, and the high expression of SNHG7 promotes the growth, migration, and invasion of bladder cancer cells [47]. In pancreatic cancer, SNHG7 is highly expressed, and the knockdown of this gene inhibits tumor progression through the miR-342-3p/ID4 axis [48]. A recent study found that SNHG7 directly binds to miR-34a and enhances migration and invasion of gastric cancer cells by inhibiting the miR-34a-Snail-EMT axis [49]. We then treated METTL3-inhibited MC-40 and AGS cells with the SNHG7 overexpression plasmid and found that SNHG7 overexpression reversed the effects of METTL3 down-regulation on cell bio-function. Furthermore, we observed that the overexpression of SNHG7 significantly enhanced the proliferation, migration, and invasion of MC-40 and AGS cells, as well as the expression of EMT marker proteins, implying that SNHG7 may be a potential oncogene for GC. In addition, the study found that SNHG7 intervention inhibited the proliferation of GC cells, which was associated with an increase in the apoptotic rate and arrest of the cell cycle at the G1/G0 phase. Regarding the underlying mechanism, p15 and p16 comprise potential targets of SNHG7 [50]. In the present study, we revealed that SNHG7 acts as a downstream target of METTL3 in the m6A depend-manner, which modulates GC onset by regulating cell proliferation, migration, and invasion. 

The toxic effects of MNNG on GC development were conclusively demonstrated by the METTL3-KO/WT transgenic mice model, and the related molecular mechanism was rigorously examined. After 18 weeks of MNNG exposure, mice unequivocally displayed a noticeable decrease in body weight growth rate, providing clear evidence of systemic toxicity, consistent with previous studies [51,52]. In our previous study, we observed that prolonged exposure to MNNG led to an increase in total m6A levels in different generations of MC cells in vitro. Therefore, we continued to investigate whether MNNG exposure affects m6A modification levels in vivo and found that MNNG exposure also induced an increase in total m6A levels in mice plasma.

The mechanism of MNNG-induced gastric toxicity was investigated from several aspects. Initially, we found that exposure to MNNG enhanced the mRNA and protein expression of METTL3, which led to an up-trend in the total m6A level. In addition, the expression levels of SNHG7 and cell proliferation indicator Ki67 were up-regulated in Mettl3-KO/WT transgenic mice exposed to MNNG. Impressively, we found that the knockout of METTL3 suppressed the MNNG exposure-induced increase in SNHG7 expression and total RNA m6A levels, suggesting that METTL3 may dominate the upward trend in SNHG7 expression in m6A modification during MNNG exposure.

The epithelial–mesenchymal transition (EMT) marks the onset of tumor metastasis, during which cells display a highly migratory and invasive mesenchymal phenotype [53]. EMT drives GC progression and metastasis, with m6A playing a crucial role in this transformative process [54,55]. This aligns with our previous findings that METTL3 influences EMT processes, especially in the context of MNNG exposure [9,13]. Previous studies have found that METTL3 may promote EMT in GC by regulating the m6A level of KLHL5, leading to the distant metastasis of gastric cancer, especially lung metastasis [56]. 

The absence of epithelial marker expression and the increase of mesenchymal marker expression is thought to indicate that the cell is undergoing EMT, during which the markers acquired include N-cadherin, Vimentin, α-SMA, Fibronectin, and Vitronectin, which together comprise key mesenchymal markers. In our present findings, cooperation between the METTL3/m6A/SNHG7 axis in EMT is seen in vivo and in vitro. In METLL3 knockdown GC cells, EMT markers N-cadherin, Vimentin, and α-SMA were significantly decreased. In contrast, the overexpression of SNHG7 partially reverted the effect of METTL3 knockdown on mesenchymal markers, such as N-cadherin, Vimentin, and α-SMA. In addition, we found that the depletion of METTL3 in METTL3-KO transgenic mice reduced levels of N-cadherin, Vimentin, α-SMA, and Snail protein while promoting increased expression of E-cadherin protein. lncRNAs could regulate gene expression through alternative splicing [57], microRNA sponging, transcription factor titration, and chromatin modification [58,59], forming the basis for the METTL3/m6A/SNHG7 axis in EMT [60,61].

Our study provides novel insights into the significance of m6A modification in environmental chemical carcinogenesis and highlights the potential of SNHG7 as a therapeutic target in GC clinical treatment. However, this study has some limitations. For instance, it did not analyze the specific types of lesions induced by MNNG, nor evaluate the degree of malignancy or pathologic classification of the lesions.

In conclusion, our study demonstrated that prolonged exposure to MNNG elevated the expression level of METTL3 and SNHG7 and promoted EMT transformation in gastric mucosal epithelial cells. The knockdown of METTL3 inhibited cell proliferation, migration, and invasion in vitro. Mechanistically, we found that METTL3 promotes the EMT process through the METTL3/m6A/SNHG7 axis in MNNG-induced GC. However, more profound mechanistic studies are required in the future.

## Figures and Tables

**Table 1:** Primer sequence of target genes.

Gene	Primer	Sequence
Ki67	Forward primer	5′-ATCATTGACCGCTCCTTTAGGT-3′
	Reverse primer	5′-GCTCGCCTTGATGGTTCCT-3′
METTL3	Forward primer	5′-CTGGGCACTTGGATTTAAGGAA-3′
	Reverse primer	5′-TGAGAGGTGGTGTAGCAACTT-3′
SNHG7	Forward primer	5′-ATGCTGACCATGCAACCCTT-3′
	Reverse primer	5′-GACATTTTGCAGAGCCGTGG-3′
GAPDH	Forward primer	5′-AGGTCGGTGTGAACGGATTTG-3′
	Reverse primer	5′-GGGGTCGTTGATGGCAACA-3′

## Data Availability

Data will be made available on request.

## Supplementary Materials

The following supporting information can be downloaded at: https://www.mdpi.com/article/10.3390/toxics12080573/s1, Figure S1: METTL3 knockdown lentiviruses and SNHG7 overexpression lentiviruses; (A) METTL3 knockdown lentiviruses; (B) SNHG7 overexpression lentiviruses; Figure S2: IHC staining for EMT marker protein after MNNG exposure. (A) IHC staining for EMT marker protein after 6 weeks of MNNG exposure. (B) IHC staining for EMT marker protein after 12 weeks of MNNG exposure. Table S1: Primer sequence of target genes.
