# Supplementary material for: METTL3-Regulated lncRNA SNHG7 Drives MNNG-Induced Epithelial–Mesenchymal Transition in Gastric Precancerous Lesions
Source: Toxics. 2024 Aug 6;12(8):573. doi: 10.3390/toxics12080573 (PMC11360688; doi:10.3390/toxics12080573)
Supplement: Supplementary file 1 [file toxics-12-00573-s001.zip › toxics-3071951-supplementary.pdf]

**Supplementary Table S1 Primer sequence of target genes**

| Gene | Primer | Sequence |
|---|---|---|
| METTL3 | Top primer | GATCCGCAAGTATGTTCACTATGAAATTCAAGAGATTTCATAGTGAACATACTTGCTTTTTTG |
| | Bottom primer | AATTCAAAAAAGCAAGTATGTTCACTATGAAATCTCTTGAATTTCATAGTGAACATACTTGCG |

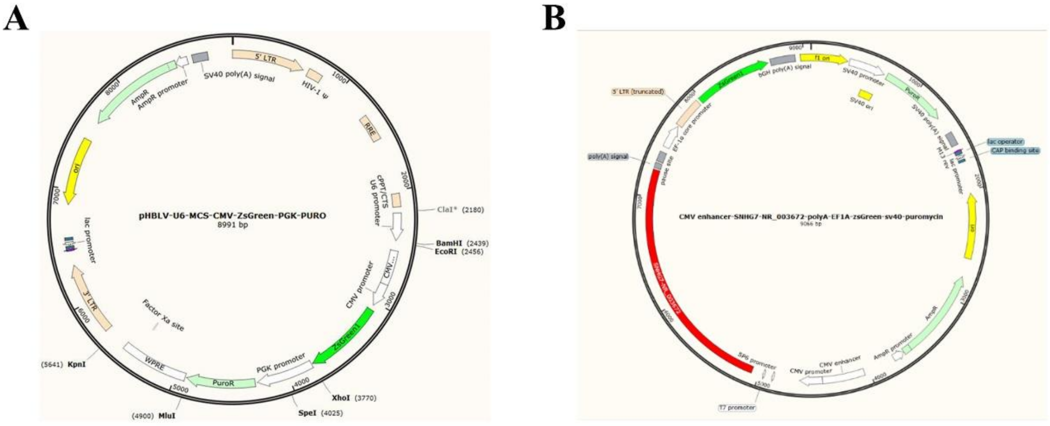


**Supplementary Figure S1.** METTL3 knockdown lentiviruses and SNHG7 overexpression lentiviruses; (A) METTL3 knockdown lentiviruses； (B) SNHG7 overexpression lentiviruses**.**

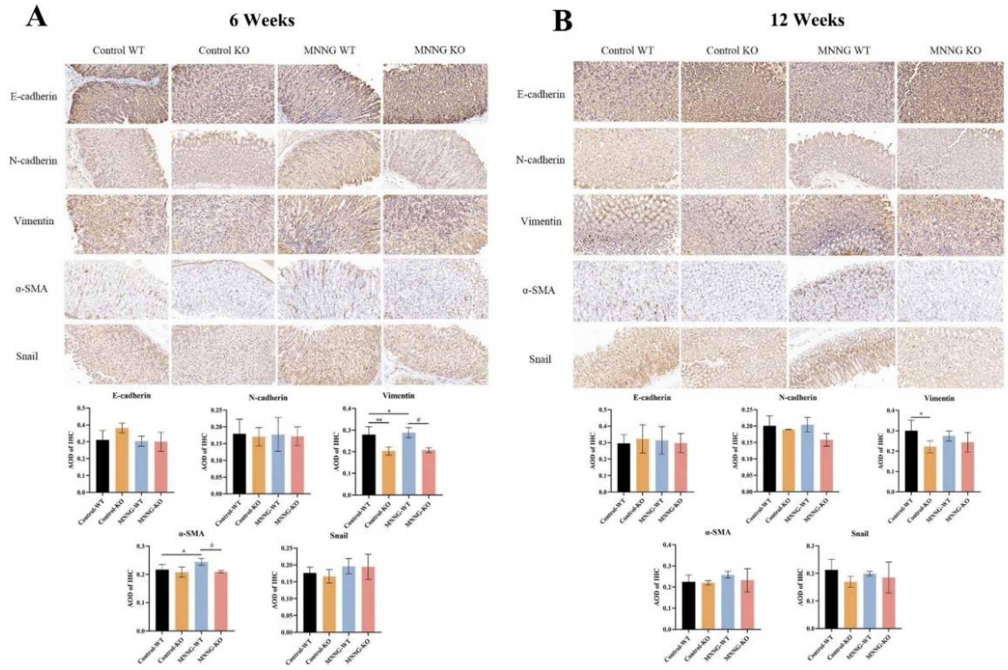


**Supplementary Figure S2.** IHC staining for EMT marker protein after MNNG exposure. (A) IHC staining for EMT marker protein after 6 weeks of MNNG exposure. (B) IHC staining for EMT marker protein after 12 weeks of MNNG exposure.
